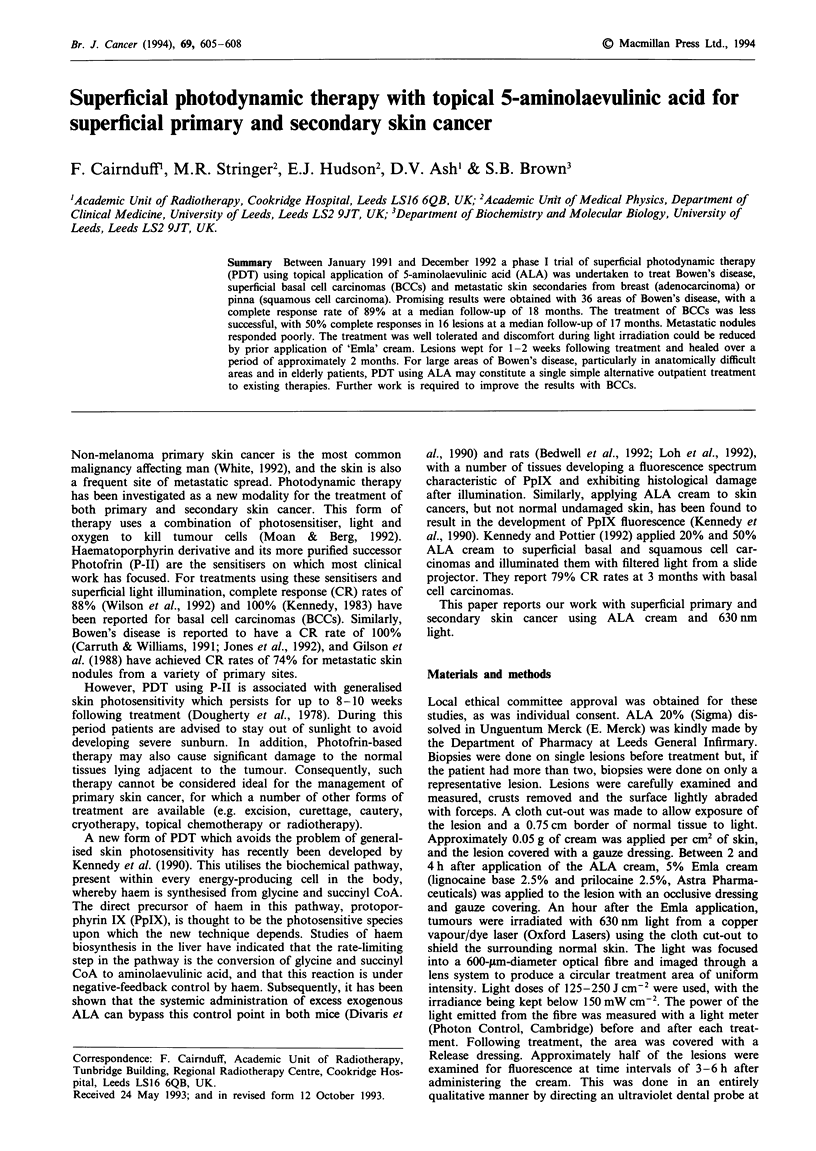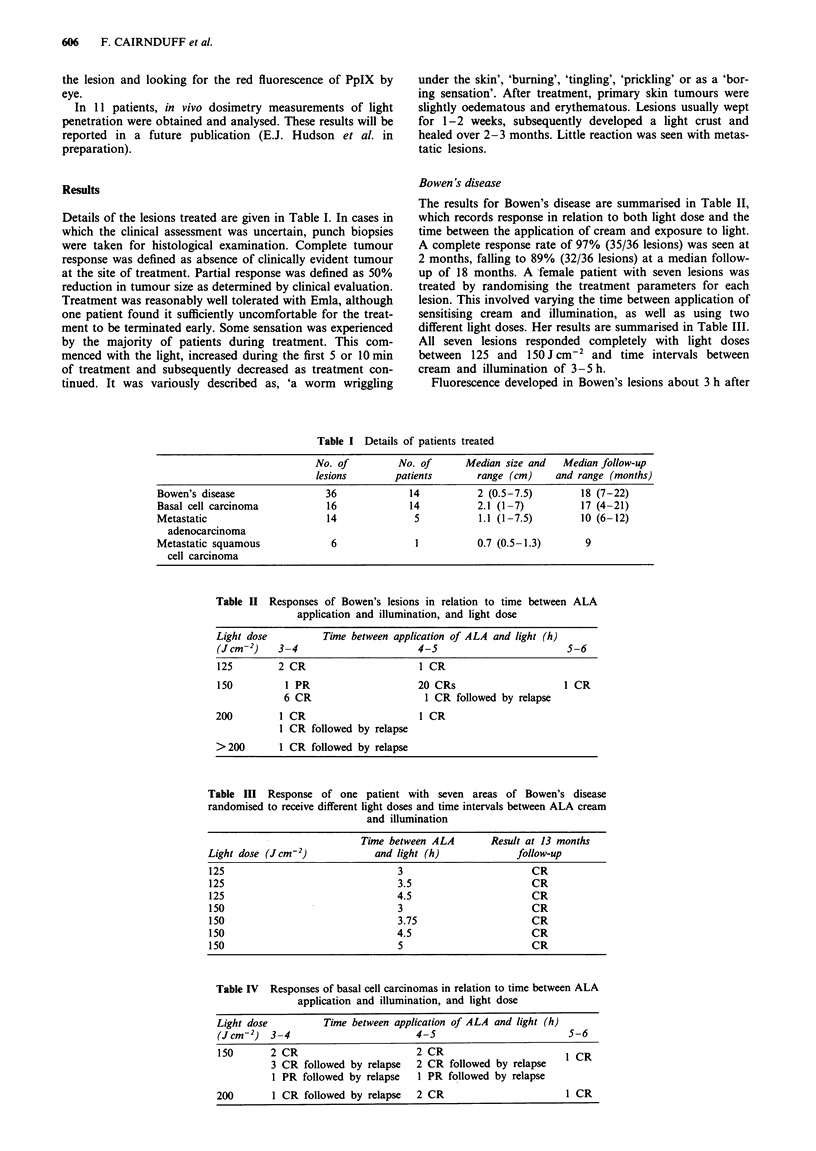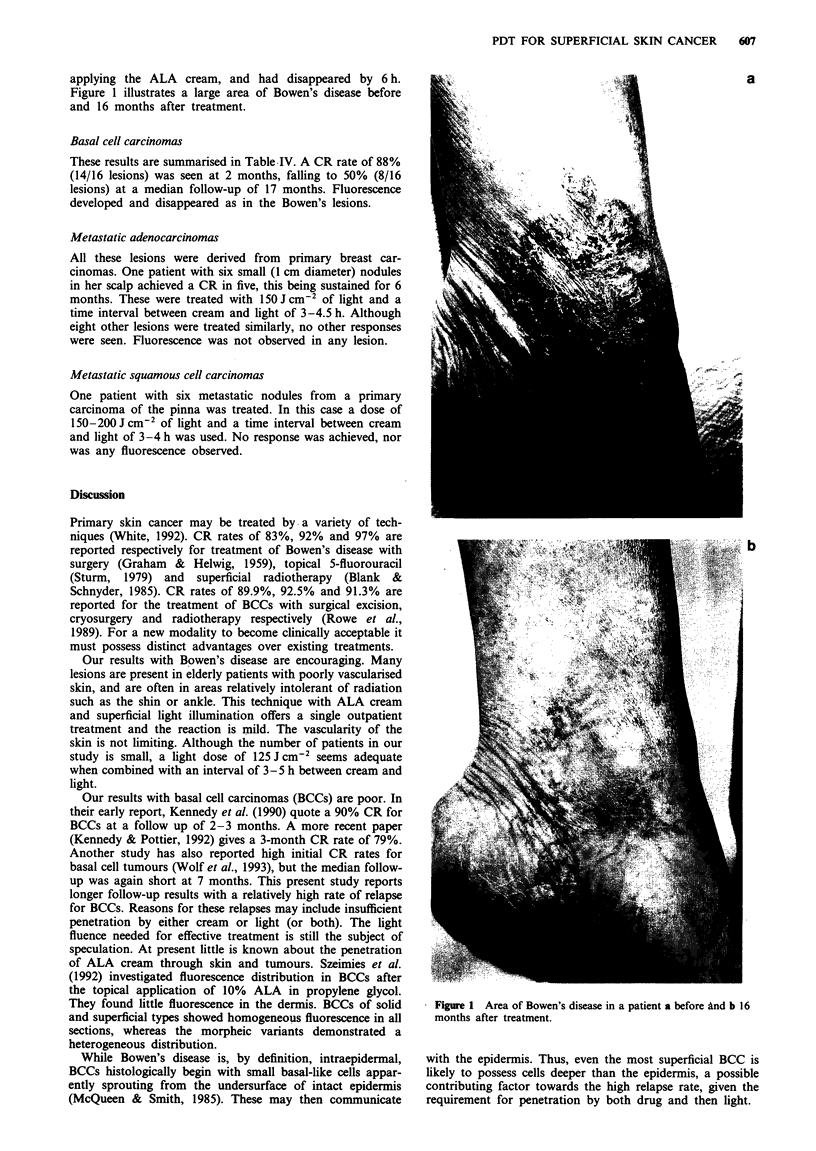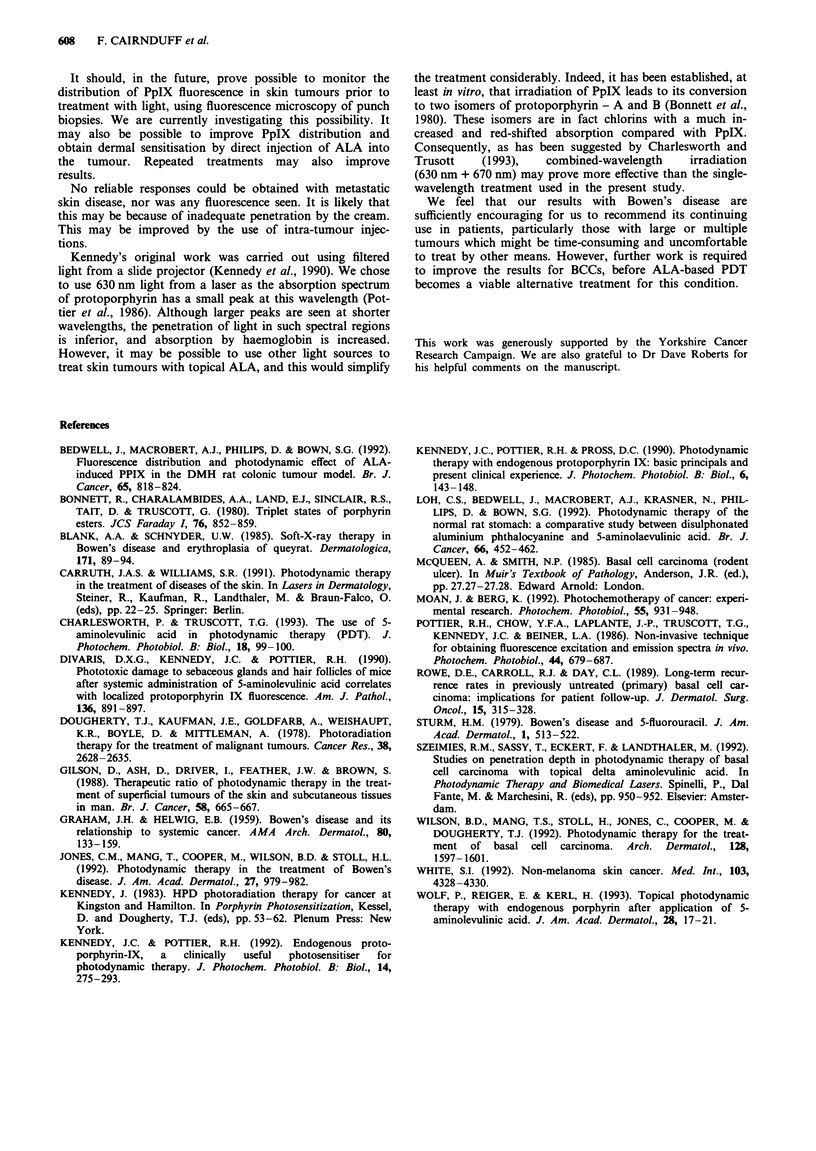# Superficial photodynamic therapy with topical 5-aminolaevulinic acid for superficial primary and secondary skin cancer.

**DOI:** 10.1038/bjc.1994.112

**Published:** 1994-03

**Authors:** F. Cairnduff, M. R. Stringer, E. J. Hudson, D. V. Ash, S. B. Brown

**Affiliations:** Academic Unit of Radiotherapy, Cookridge Hospital, Leeds, UK.

## Abstract

**Images:**


Br. J. Cancer (1994), 69, 605-608 © Macmillan Press Ltd., 1994

### Superficial photodynamic therapy with topical 5-aminolaevulinic acid for superficial primary and secondary skin cancer

F. Cairnduff[1], M.R. Stringer[2], E.J. Hudson[2], D.V. Ash[1] & S.B. Brown[3]

[1]*Academic Unit of Radiotherapy, Cookridge Hospital, Leeds LS16 6QB, UK;* [2]*Academic Unit of Medical Physics, Department of Clinical Medicine, University of Leeds, Leeds LS2 9JT, UK;* [3]*Department of Biochemistry and Molecular Biology, University of Leeds, Leeds LS2 9JT, UK.*

**Summary** Between January 1991 and December 1992 a phase I trial of superficial photodynamic therapy (PDT) using topical application of 5-aminolaevulinic acid (ALA) was undertaken to treat Bowen's disease, superficial basal cell carcinomas (BCCs) and metastatic skin secondaries from breast (adenocarcinoma) or pinna (squamous cell carcinoma). Promising results were obtained with 36 areas of Bowen's disease, with a complete response rate of 89% at a median follow-up of 18 months. The treatment of BCCs was less successful, with 50% complete responses in 16 lesions at a median follow-up of 17 months. Metastatic nodules responded poorly. The treatment was well tolerated and discomfort during light irradiation could be reduced by prior application of 'Emla' cream. Lesions wept for 1-2 weeks following treatment and healed over a period of approximately 2 months. For large areas of Bowen's disease, particularly in anatomically difficult areas and in elderly patients, PDT using ALA may constitute a single simple alternative outpatient treatment to existing therapies. Further work is required to improve the results with BCCs.

Non-melanoma primary skin cancer is the most common malignancy affecting man (White, 1992), and the skin is also a frequent site of metastatic spread. Photodynamic therapy has been investigated as a new modality for the treatment of both primary and secondary skin cancer. This form of therapy uses a combination of photosensitiser, light and oxygen to kill tumour cells (Moan & Berg, 1992). Haematoporphyrin derivative and its more purified successor Photofrin (P-II) are the sensitisers on which most clinical work has focused. For treatments using these sensitisers and superficial light illumination, complete response (CR) rates of 88% (Wilson *et al.*, 1992) and 100% (Kennedy, 1983) have been reported for basal cell carcinomas (BCCs). Similarly, Bowen's disease is reported to have a CR rate of 100% (Carruth & Williams, 1991; Jones *et al.*, 1992), and Gilson *et al.* (1988) have achieved CR rates of 74% for metastatic skin nodules from a variety of primary sites.

However, PDT using P-II is associated with generalised skin photosensitivity which persists for up to 8-10 weeks following treatment (Dougherty *et al.*, 1978). During this period patients are advised to stay out of sunlight to avoid developing severe sunburn. In addition, Photofrin-based therapy may also cause significant damage to the normal tissues lying adjacent to the tumour. Consequently, such therapy cannot be considered ideal for the management of primary skin cancer, for which a number of other forms of treatment are available (e.g. excision, curettage, cautery, cryotherapy, topical chemotherapy or radiotherapy).

A new form of PDT which avoids the problem of generalised skin photosensitivity has recently been developed by Kennedy *et al.* (1990). This utilises the biochemical pathway, present within every energy-producing cell in the body, whereby haem is synthesised from glycine and succinyl CoA. The direct precursor of haem in this pathway, protoporphyrin IX (PpIX), is thought to be the photosensitive species upon which the new technique depends. Studies of haem biosynthesis in the liver have indicated that the rate-limiting step in the pathway is the conversion of glycine and succinyl CoA to aminolaevulinic acid, and that this reaction is under negative-feedback control by haem. Subsequently, it has been shown that the systemic administration of excess exogenous ALA can bypass this control point in both mice (Divaris *et al.*, 1990) and rats (Bedwell *et al.*, 1992; Loh *et al.*, 1992), with a number of tissues developing a fluorescence spectrum characteristic of PpIX and exhibiting histological damage after illumination. Similarly, applying ALA cream to skin cancers, but not normal undamaged skin, has been found to result in the development of PpIX fluorescence (Kennedy *et al.*, 1990). Kennedy and Pottier (1992) applied 20% and 50% ALA cream to superficial basal and squamous cell carcinomas and illuminated them with filtered light from a slide projector. They report 79% CR rates at 3 months with basal cell carcinomas.

This paper reports our work with superficial primary and secondary skin cancer using ALA cream and 630 nm light.

### Materials and methods

Local ethical committee approval was obtained for these studies, as was individual consent. ALA 20% (Sigma) dissolved in Unguentum Merck (E. Merck) was kindly made by the Department of Pharmacy at Leeds General Infirmary. Biopsies were done on single lesions before treatment but, if the patient had more than two, biopsies were done on only a representative lesion. Lesions were carefully examined and measured, crusts removed and the surface lightly abraded with forceps. A cloth cut-out was made to allow exposure of the lesion and a 0.75 cm border of normal tissue to light. Approximately 0.05 g of cream was applied per $cm^2$ of skin, and the lesion covered with a gauze dressing. Between 2 and 4 h after application of the ALA cream, 5% Emla cream (lignocaine base 2.5% and prilocaine 2.5%, Astra Pharmaceuticals) was applied to the lesion with an occlusive dressing and gauze covering. An hour after the Emla application, tumours were irradiated with 630 nm light from a copper vapour/dye laser (Oxford Lasers) using the cloth cut-out to shield the surrounding normal skin. The light was focused into a 600-μm-diameter optical fibre and imaged through a lens system to produce a circular treatment area of uniform intensity. Light doses of 125-250 J $cm^{-2}$ were used, with the irradiance being kept below 150 mW $cm^{-2}$. The power of the light emitted from the fibre was measured with a light meter (Photon Control, Cambridge) before and after each treatment. Following treatment, the area was covered with a Release dressing. Approximately half of the lesions were examined for fluorescence at time intervals of 3-6 h after administering the cream. This was done in an entirely qualitative manner by directing an ultraviolet dental probe at

Correspondence: F. Cairnduff, Academic Unit of Radiotherapy, Tunbridge Building, Regional Radiotherapy Centre, Cookridge Hospital, Leeds LS16 6QB, UK.
Received 24 May 1993; and in revised form 12 October 1993.

the lesion and looking for the red fluorescence of PpIX by eye.

In 11 patients, *in vivo* dosimetry measurements of light penetration were obtained and analysed. These results will be reported in a future publication (E.J. Hudson *et al.* in preparation).

### Results

Details of the lesions treated are given in Table I. In cases in which the clinical assessment was uncertain, punch biopsies were taken for histological examination. Complete tumour response was defined as absence of clinically evident tumour at the site of treatment. Partial response was defined as 50% reduction in tumour size as determined by clinical evaluation. Treatment was reasonably well tolerated with Emla, although one patient found it sufficiently uncomfortable for the treatment to be terminated early. Some sensation was experienced by the majority of patients during treatment. This commenced with the light, increased during the first 5 or 10 min of treatment and subsequently decreased as treatment continued. It was variously described as, 'a worm wriggling under the skin', 'burning', 'tingling', 'prickling' or as a 'boring sensation'. After treatment, primary skin tumours were slightly oedematous and erythematous. Lesions usually wept for 1–2 weeks, subsequently developed a light crust and healed over 2–3 months. Little reaction was seen with metastatic lesions.

### *Bowen's disease*

The results for Bowen's disease are summarised in Table II, which records response in relation to both light dose and the time between the application of cream and exposure to light. A complete response rate of 97% (35/36 lesions) was seen at 2 months, falling to 89% (32/36 lesions) at a median follow-up of 18 months. A female patient with seven lesions was treated by randomising the treatment parameters for each lesion. This involved varying the time between application of sensitising cream and illumination, as well as using two different light doses. Her results are summarised in Table III. All seven lesions responded completely with light doses between 125 and 150 J $cm^{-2}$ and time intervals between cream and illumination of 3–5 h.

Fluorescence developed in Bowen's lesions about 3 h after

**Table I** Details of patients treated

| | *No. of lesions* | *No. of patients* | *Median size and range (cm)* | *Median follow-up and range (months)* |
|---|---|---|---|---|
| Bowen's disease | 36 | 14 | 2 (0.5–7.5) | 18 (7–22) |
| Basal cell carcinoma | 16 | 14 | 2.1 (1–7) | 17 (4–21) |
| Metastatic adenocarcinoma | 14 | 5 | 1.1 (1–7.5) | 10 (6–12) |
| Metastatic squamous cell carcinoma | 6 | 1 | 0.7 (0.5–1.3) | 9 |

**Table II** Responses of Bowen's lesions in relation to time between ALA application and illumination, and light dose

| *Light dose* | *Time between application of ALA and light (h)* | | |
|---|---|---|---|
| *(J $cm^{-2}$)* | *3–4* | *4–5* | *5–6* |
| 125 | 2 CR | 1 CR | |
| 150 | 1 PR<br>6 CR | 20 CRs<br>1 CR followed by relapse | 1 CR |
| 200 | 1 CR<br>1 CR followed by relapse | 1 CR | |
| >200 | 1 CR followed by relapse | | |

**Table III** Response of one patient with seven areas of Bowen's disease randomised to receive different light doses and time intervals between ALA cream and illumination

| *Light dose (J $cm^{-2}$)* | *Time between ALA and light (h)* | *Result at 13 months follow-up* |
|---|---|---|
| 125 | 3 | CR |
| 125 | 3.5 | CR |
| 125 | 4.5 | CR |
| 150 | 3 | CR |
| 150 | 3.75 | CR |
| 150 | 4.5 | CR |
| 150 | 5 | CR |

**Table IV** Responses of basal cell carcinomas in relation to time between ALA application and illumination, and light dose

| *Light dose* | *Time between application of ALA and light (h)* | | |
|---|---|---|---|
| *(J $cm^{-2}$)* | *3–4* | *4–5* | *5–6* |
| 150 | 2 CR<br>3 CR followed by relapse<br>1 PR followed by relapse | 2 CR<br>2 CR followed by relapse<br>1 PR followed by relapse | 1 CR |
| 200 | 1 CR followed by relapse | 2 CR | 1 CR |

applying the ALA cream, and had disappeared by 6 h. Figure 1 illustrates a large area of Bowen's disease before and 16 months after treatment.

*Basal cell carcinomas*

These results are summarised in Table IV. A CR rate of 88% (14/16 lesions) was seen at 2 months, falling to 50% (8/16 lesions) at a median follow-up of 17 months. Fluorescence developed and disappeared as in the Bowen's lesions.

*Metastatic adenocarcinomas*

All these lesions were derived from primary breast carcinomas. One patient with six small (1 cm diameter) nodules in her scalp achieved a CR in five, this being sustained for 6 months. These were treated with 150 J $cm^{-2}$ of light and a time interval between cream and light of 3–4.5 h. Although eight other lesions were treated similarly, no other responses were seen. Fluorescence was not observed in any lesion.

*Metastatic squamous cell carcinomas*

One patient with six metastatic nodules from a primary carcinoma of the pinna was treated. In this case a dose of 150–200 J $cm^{-2}$ of light and a time interval between cream and light of 3–4 h was used. No response was achieved, nor was any fluorescence observed.

### Discussion

Primary skin cancer may be treated by a variety of techniques (White, 1992). CR rates of 83%, 92% and 97% are reported respectively for treatment of Bowen's disease with surgery (Graham & Helwig, 1959), topical 5-fluorouracil (Sturm, 1979) and superficial radiotherapy (Blank & Schnyder, 1985). CR rates of 89.9%, 92.5% and 91.3% are reported for the treatment of BCCs with surgical excision, cryosurgery and radiotherapy respectively (Rowe *et al.*, 1989). For a new modality to become clinically acceptable it must possess distinct advantages over existing treatments.

Our results with Bowen's disease are encouraging. Many lesions are present in elderly patients with poorly vascularised skin, and are often in areas relatively intolerant of radiation such as the shin or ankle. This technique with ALA cream and superficial light illumination offers a single outpatient treatment and the reaction is mild. The vascularity of the skin is not limiting. Although the number of patients in our study is small, a light dose of 125 J $cm^{-2}$ seems adequate when combined with an interval of 3–5 h between cream and light.

Our results with basal cell carcinomas (BCCs) are poor. In their early report, Kennedy *et al.* (1990) quote a 90% CR for BCCs at a follow up of 2–3 months. A more recent paper (Kennedy & Pottier, 1992) gives a 3-month CR rate of 79%. Another study has also reported high initial CR rates for basal cell tumours (Wolf *et al.*, 1993), but the median follow-up was again short at 7 months. This present study reports longer follow-up results with a relatively high rate of relapse for BCCs. Reasons for these relapses may include insufficient penetration by either cream or light (or both). The light fluence needed for effective treatment is still the subject of speculation. At present little is known about the penetration of ALA cream through skin and tumours. Szeimies *et al.* (1992) investigated fluorescence distribution in BCCs after the topical application of 10% ALA in propylene glycol. They found little fluorescence in the dermis. BCCs of solid and superficial types showed homogeneous fluorescence in all sections, whereas the morpheic variants demonstrated a heterogeneous distribution.

While Bowen's disease is, by definition, intraepidermal, BCCs histologically begin with small basal-like cells apparently sprouting from the undersurface of intact epidermis (McQueen & Smith, 1985). These may then communicate with the epidermis. Thus, even the most superficial BCC is likely to possess cells deeper than the epidermis, a possible contributing factor towards the high relapse rate, given the requirement for penetration by both drug and then light.

**Figure 1** Area of Bowen's disease in a patient **a** before and **b** 16 months after treatment.

It should, in the future, prove possible to monitor the distribution of PpIX fluorescence in skin tumours prior to treatment with light, using fluorescence microscopy of punch biopsies. We are currently investigating this possibility. It may also be possible to improve PpIX distribution and obtain dermal sensitisation by direct injection of ALA into the tumour. Repeated treatments may also improve results.

No reliable responses could be obtained with metastatic skin disease, nor was any fluorescence seen. It is likely that this may be because of inadequate penetration by the cream. This may be improved by the use of intra-tumour injections.

Kennedy's original work was carried out using filtered light from a slide projector (Kennedy *et al.*, 1990). We chose to use 630 nm light from a laser as the absorption spectrum of protoporphyrin has a small peak at this wavelength (Pottier *et al.*, 1986). Although larger peaks are seen at shorter wavelengths, the penetration of light in such spectral regions is inferior, and absorption by haemoglobin is increased. However, it may be possible to use other light sources to treat skin tumours with topical ALA, and this would simplify the treatment considerably. Indeed, it has been established, at least *in vitro*, that irradiation of PpIX leads to its conversion to two isomers of protoporphyrin – A and B (Bonnett *et al.*, 1980). These isomers are in fact chlorins with a much increased and red-shifted absorption compared with PpIX. Consequently, as has been suggested by Charlesworth and Trusott (1993), combined-wavelength irradiation (630 nm + 670 nm) may prove more effective than the single-wavelength treatment used in the present study.

We feel that our results with Bowen's disease are sufficiently encouraging for us to recommend its continuing use in patients, particularly those with large or multiple tumours which might be time-consuming and uncomfortable to treat by other means. However, further work is required to improve the results for BCCs, before ALA-based PDT becomes a viable alternative treatment for this condition.

This work was generously supported by the Yorkshire Cancer Research Campaign. We are also grateful to Dr Dave Roberts for his helpful comments on the manuscript.

### References

BEDWELL, J., MACROBERT, A.J., PHILIPS, D. & BOWN, S.G. (1992). Fluorescence distribution and photodynamic effect of ALA-induced PPIX in the DMH rat colonic tumour model. *Br. J. Cancer*, **65**, 818–824.

BONNETT, R., CHARALAMBIDES, A.A., LAND, E.J., SINCLAIR, R.S., TAIT, D. & TRUSCOTT, G. (1980). Triplet states of porphyrin esters. *JCS Faraday I*, **76**, 852–859.

BLANK, A.A. & SCHNYDER, U.W. (1985). Soft-X-ray therapy in Bowen's disease and erythroplasia of queyrat. *Dermatologica*, **171**, 89–94.

CARRUTH, J.A.S. & WILLIAMS, S.R. (1991). Photodynamic therapy in the treatment of diseases of the skin. In *Lasers in Dermatology*, Steiner, R., Kaufman, R., Landthaler, M. & Braun-Falco, O. (eds), pp. 22–25. Springer: Berlin.

CHARLESWORTH, P. & TRUSCOTT, T.G. (1993). The use of 5-aminolevulinic acid in photodynamic therapy (PDT). *J. Photochem. Photobiol. B: Biol.*, **18**, 99–100.

DIVARIS, D.X.G., KENNEDY, J.C. & POTTIER, R.H. (1990). Phototoxic damage to sebaceous glands and hair follicles of mice after systemic administration of 5-aminolevulinic acid correlates with localized protoporphyrin IX fluorescence. *Am. J. Pathol.*, **136**, 891–897.

DOUGHERTY, T.J., KAUFMAN, J.E., GOLDFARB, A., WEISHAUPT, K.R., BOYLE, D. & MITTLEMAN, A. (1978). Photoradiation therapy for the treatment of malignant tumours. *Cancer Res.*, **38**, 2628–2635.

GILSON, D., ASH, D., DRIVER, I., FEATHER, J.W. & BROWN, S. (1988). Therapeutic ratio of photodynamic therapy in the treatment of superficial tumours of the skin and subcutaneous tissues in man. *Br. J. Cancer*, **58**, 665–667.

GRAHAM, J.H. & HELWIG, E.B. (1959). Bowen's disease and its relationship to systemic cancer. *AMA Arch. Dermatol.*, **80**, 133–159.

JONES, C.M., MANG, T., COOPER, M., WILSON, B.D. & STOLL, H.L. (1992). Photodynamic therapy in the treatment of Bowen's disease. *J. Am. Acad. Dermatol.*, **27**, 979–982.

KENNEDY, J. (1983). HPD photoradiation therapy for cancer at Kingston and Hamilton. In *Porphyrin Photosensitization*, Kessel, D. and Dougherty, T.J. (eds), pp. 53–62. Plenum Press: New York.

KENNEDY, J.C. & POTTIER, R.H. (1992). Endogenous protoporphyrin-IX, a clinically useful photosensitiser for photodynamic therapy. *J. Photochem. Photobiol. B: Biol.*, **14**, 275–293.

KENNEDY, J.C., POTTIER, R.H. & PROSS, D.C. (1990). Photodynamic therapy with endogenous protoporphyrin IX: basic principals and present clinical experience. *J. Photochem. Photobiol. B: Biol.*, **6**, 143–148.

LOH, C.S., BEDWELL, J., MACROBERT, A.J., KRASNER, N., PHILLIPS, D. & BOWN, S.G. (1992). Photodynamic therapy of the normal rat stomach: a comparative study between disulphonated aluminium phthalocyanine and 5-aminolaevulinic acid. *Br. J. Cancer*, **66**, 452–462.

MCQUEEN, A. & SMITH, N.P. (1985). Basal cell carcinoma (rodent ulcer). In *Muir's Textbook of Pathology*, Anderson, J.R. (ed.), pp. 27.27–27.28. Edward Arnold: London.

MOAN, J. & BERG, K. (1992). Photochemotherapy of cancer: experimental research. *Photochem. Photobiol.*, **55**, 931–948.

POTTIER, R.H., CHOW, Y.F.A., LAPLANTE, J.-P., TRUSCOTT, T.G., KENNEDY, J.C. & BEINER, L.A. (1986). Non-invasive technique for obtaining fluorescence excitation and emission spectra *in vivo*. *Photochem. Photobiol.*, **44**, 679–687.

ROWE, D.E., CARROLL, R.J. & DAY, C.L. (1989). Long-term recurrence rates in previously untreated (primary) basal cell carcinoma: implications for patient follow-up. *J. Dermatol. Surg. Oncol.*, **15**, 315–328.

STURM, H.M. (1979). Bowen's disease and 5-fluorouracil. *J. Am. Acad. Dermatol.*, **1**, 513–522.

SZEIMIES, R.M., SASSY, T., ECKERT, F. & LANDTHALER, M. (1992). Studies on penetration depth in photodynamic therapy of basal cell carcinoma with topical delta aminolevulinic acid. In *Photodynamic Therapy and Biomedical Lasers*. Spinelli, P., Dal Fante, M. & Marchesini, R. (eds), pp. 950–952. Elsevier: Amsterdam.

WILSON, B.D., MANG, T.S., STOLL, H., JONES, C., COOPER, M. & DOUGHERTY, T.J. (1992). Photodynamic therapy for the treatment of basal cell carcinoma. *Arch. Dermatol.*, **128**, 1597–1601.

WHITE, S.I. (1992). Non-melanoma skin cancer. *Med. Int.*, **103**, 4328–4330.

WOLF, P., REIGER, E. & KERL, H. (1993). Topical photodynamic therapy with endogenous porphyrin after application of 5-aminolevulinic acid. *J. Am. Acad. Dermatol.*, **28**, 17–21.